# Oxygen-augmented erythrocyte-liposome hybrids to overcome paclitaxel resistance in triple-negative breast cancer metastasis via enhanced ferroptosis

**DOI:** 10.1016/j.mtbio.2026.103705

**Published:** 2026-09-23

**Authors:** Xunyi You, Kehui Zhu, Wanjing Li, Hong Wang, Rui Zhong, Shen Li, Jiaxin Liu, Yee Shan Wong, Subbu S. Venkatraman, Ye Cao

**Affiliations:** a Institute of Blood Transfusion, Chinese Academy of Medical Sciences & Peking Union Medical College, Chengdu, Sichuan Province, China; b Department of Blood Transfusion, Sichuan Clinical Research Center for Cancer, Sichuan Cancer Hospital & Institute, Sichuan Cancer Center, University of Electronic Science and Technology of China, Chengdu, China; c Biomedical Engineering, School of Engineering, Temasek Polytechnic, Singapore, Singapore; d School of Materials Science and Engineering, National University of Singapore, Singapore, Singapore; e Singapore Eye Research Institute, Singapore, Singapore; f School of Materials Science and Engineering, Nanyang Technological University, Singapore, Singapore

**Keywords:** Liposome, Hemoglobin, Red blood cell hitchhiking, Chemoresistance, Hypoxia

## Abstract

Paclitaxel (PTX) resistance in triple-negative breast cancer (TNBC) is closely associated with ATP-binding cassette (ABC) transporters-medicated drug efflux and hypoxia-driven upregulation of hypoxia-inducible factor-1α (HIF-1α), which together promote tumor progression and metastasis. Although blood transfusion or hyperbaric oxygen therapy can partially alleviate systemic hypoxia, neither approach effectively relieves chemoresistance driven by intratumoral hypoxia. To address this challenge, we have developed a biomimetic hybrid delivery system (red blood cell hitchhiking-liposomes encapsulate hemoglobin and paclitaxel, RBC-LEHP) that utilizes red blood cell-hitchhiking technology along with pH-responsive liposomes, to co-deliver hemoglobin (Hb) and PTX to overcome hypoxia-associated chemoresistance. This system achieves dual-stage targeting: passive sequestration in pulmonary metastatic lesions through erythrocyte hitchhiking and active tumor internalization mediated by pH-sensitive DOPE phospholipids. Our study revealed that RBC-LEHP effectively alleviated the hypoxia microenvironment through Hb-mediated oxygenation upon tumor cellular internalization and downregulated HIF-1α and glutathione peroxidase 4 (GPX4), while simultaneously enhancing reactive oxygen species (ROS)-mediated lipid peroxidation to induce ferroptosis. This amplified oxidative stress further triggered mitochondrial dysfunction, suppressed P-glycoprotein (P-gp)-mediated PTX efflux, reversed PTX resistance, and promoted apoptotic cell death. *In vivo* results confirmed that RBC-LEHP-mediated therapeutic cascade of “lung targeted delivery → hypoxia alleviation → ferroptosis/chemotherapy → tumor suppression” significantly enhanced antitumor efficacy against TNBC. This biomimetic platform provides a promising strategy for overcoming physiological barriers and chemoresistance in metastatic TNBC therapy.

## Highlights

• A RBC-hitchhiking liposome with pH-responsive DOPE achieves active and passive lung metastasis targeting.
• Hemoglobin alleviates tumor hypoxia, downregulates HIF-1α and P-gp, and triggers ferroptosis to reverse chemoresistance.
• Co-encapsulated Hb-PTX liposome suppresses lung metastasis in 4T1 mice.

## Graphical abstract

**Figure:**
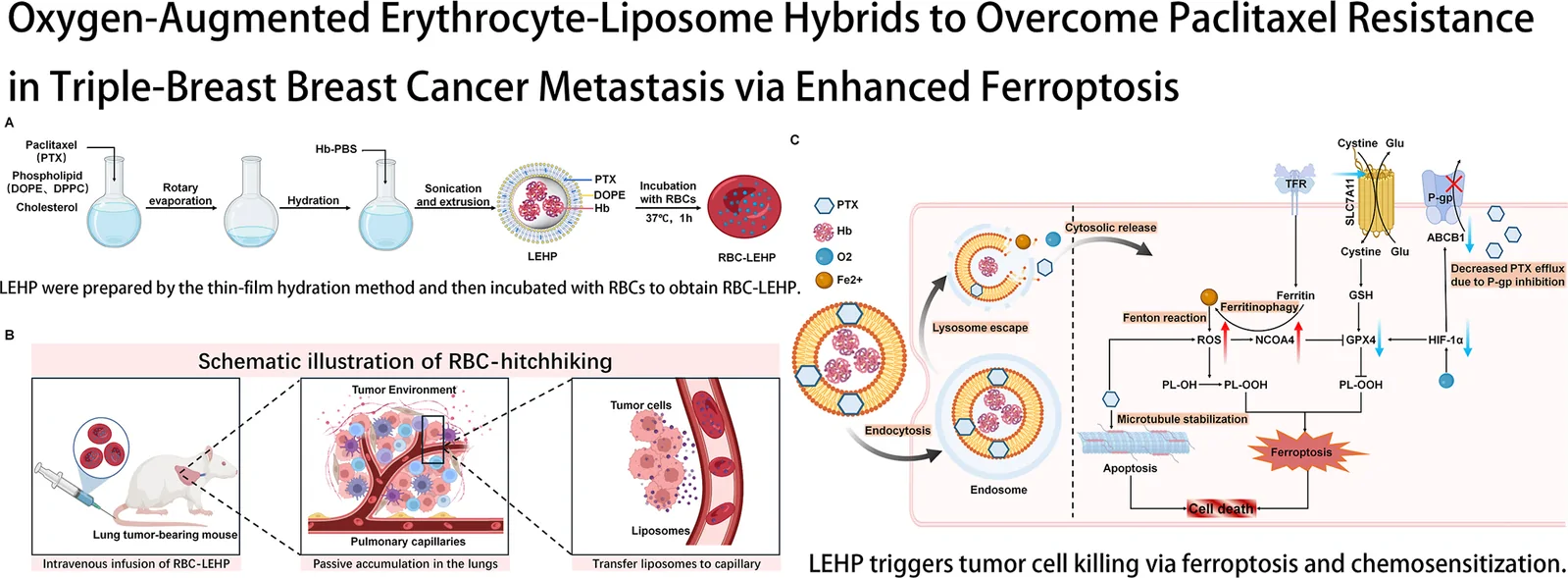

## 1 Introduction

Triple-negative breast cancer (TNBC) remains the most recalcitrant breast cancer subtype, characterized by aggressive proliferation, early metastatic dissemination and poor prognosis [1]. Lung metastasis is the primary driver of TNBC-related mortality [2], yet current chemotherapeutic interventions are severely hindered by two interlocking barriers [3]:insufficient drug accumulation in lung metastatic lesions and the development of multidrug resistance (MDR).

At the molecular level, the pronounced hypoxic microenvironment of TNBC solid tumors acts as a catalyst for MDR. Hypoxia stabilizes hypoxia-inducible factor-1α (HIF-1α), which directly orchestrates the upregulation of ATP-binding cassette (ABC) transporters, most notably P-glycoprotein (P-gp). This HIF-1α/P-gp signaling axis facilitates the active efflux of cytotoxic agents - including paclitaxel, thereby compromising therapeutic efficacy. For example, Nath et al. elucidated that HIF-1α is significantly overexpressed in chemoresistant tissues across multiple malignancies and directly upregulates the transcription of *ABCB1* (encoding P-gp), *ABCC1* (encoding MRP1), and *ABCG2* (encoding BCRP) by binding to hypoxia response elements in the promoter regions, thereby establishing a direct molecular link between hypoxia and MDR [4]. Subsequent work by Feng et al. showed that pharmacologically targeting the HIF-1α/P-gp axis—via inhibition of CRM1 with Selinexor—restores HIF-1α degradation, reduces P-gp expression, and reverses hypoxia-driven chemoresistance both *in vitro* and *in vivo* [5]. While pharmacological inhibition of this axis shows promise, achieving localized reversal of hypoxia within the metastatic niche remains a significant challenge [6,7].

Hypoxia also stimulates angiogenesis in tumor, which is essential for tumor growth [8]. This process can help tumor to obtain oxygen and nutrients, leading to the fast and unnormal vascularization inside the tumor [9]. These newly formed tumor blood vessels are irregularly shaped, having an incomplete hierarchical structure, and functional abnormalities. RBCs can enter the tumor vessels, but their circulation is severely impaired, irregular, and often dysfunctional. Previous studies proved that oxygen can only diffuse 100∼200 μm from a functional blood vessel, which implied that the center of the tumor was highly hypoxia [10]. Although blood transfusion or hyperbaric oxygen therapy can partially alleviate systemic hypoxia, neither approach effectively relieves intratumoral hypoxia-driven chemoresistance. Hemoglobin-based oxygen carriers (HBOCs) has been developed to alleviate tumor hypoxia. Hemoglobin (Hb), a natural endogenous oxygen carrier, offers dual-action potential: directly alleviate tumor hypoxia through oxygen delivery and catalyze the generation of *in situ* reactive oxygen species (ROS) [11]. However, the clinical utility of “naked” Hb is restricted by rapid oxidative degradation, dose-dependent cytotoxicity, and systemic pro-inflammatory effects. While liposome-encapsulated hemoglobin (LEH) improves stability [12], conventional formulations lack specificity, often sequestering in the liver and spleen or causing non-specific oxygenation. Enhancing the spatial precision of Hb delivery is therefore critical to minimizing off-target toxicity while maximizing its ability to sensitize tumors to chemotherapy.

Beyond hypoxia-driven molecular resistance, physiological delivery barriers persist. Most nanoparticle-based drug delivery system (DDS) rely on the enhanced permeability and retention (EPR) effect for tumor accumulation, which is highly heterogeneous in metastatic lesions. [13,14]. A retrospective study reported that only 0.6∼0.8% of the injected dose of liposomal drug formulations reached the patient tumors tissue via EPR effect [15], so current liposomal DDS still suffered from low targeting ability and off-target toxicity. Unlike conventional nanoparticle DDS, red blood cell (RBC) hitchhiking (RH) has been developed to enhance tumor-targeting ability, extend blood circulation life and reduce their off-target toxicity. The nanocarriers are loosely attached to the RBC surface (without internalization) through weak, reversible interactions (e.g. van der Waals forcers, electrostatic interactions). Upon intravenous administration, the RBC-nanocarrier DDS squeeze through the narrow capillaries, shear forces dislodge the nanoparticles from RBCs surface to the capillary. Nanoparticles are mechanically transferred to the capillaries endothelial cells and dramatically increase nanoparticle accumulation in the first capillary-rich organ downstream of the injection site. Our pervious publication have demonstrated that combining liposomes with RBC hitchhiking significantly increased both the pulmonary accumulation and the blood half-life of the liposomes [16]. Thus, RH offers a promising target therapy approach for pulmonary related diseases.

In this study, we present a lung-targeting, erythrocyte-liposome hybrid system (RBC-LEHP) designed to reverse PTX resistance by synergizing hypoxia alleviation with Hb-mediated ferroptosis (Fig. 1). By integrating pH-sensitive DOPE phospholipids with RBC-hitchhiking technology, the RBC-LEHP system achieves superior accumulation within pulmonary TNBC metastases. Upon internalization, the encapsulated Hb alleviates hypoxia and downregulates the HIF-1α/P-gp axis, while simultaneously driving ROS-dependent lipid peroxidation to trigger ferroptosis. This integrated “targeted delivery → hypoxia alleviation → ferroptosis induction” cascade offers a comprehensive biomimetic approach to eradicating resistant TNBC lung metastases.

**Fig. 1:**
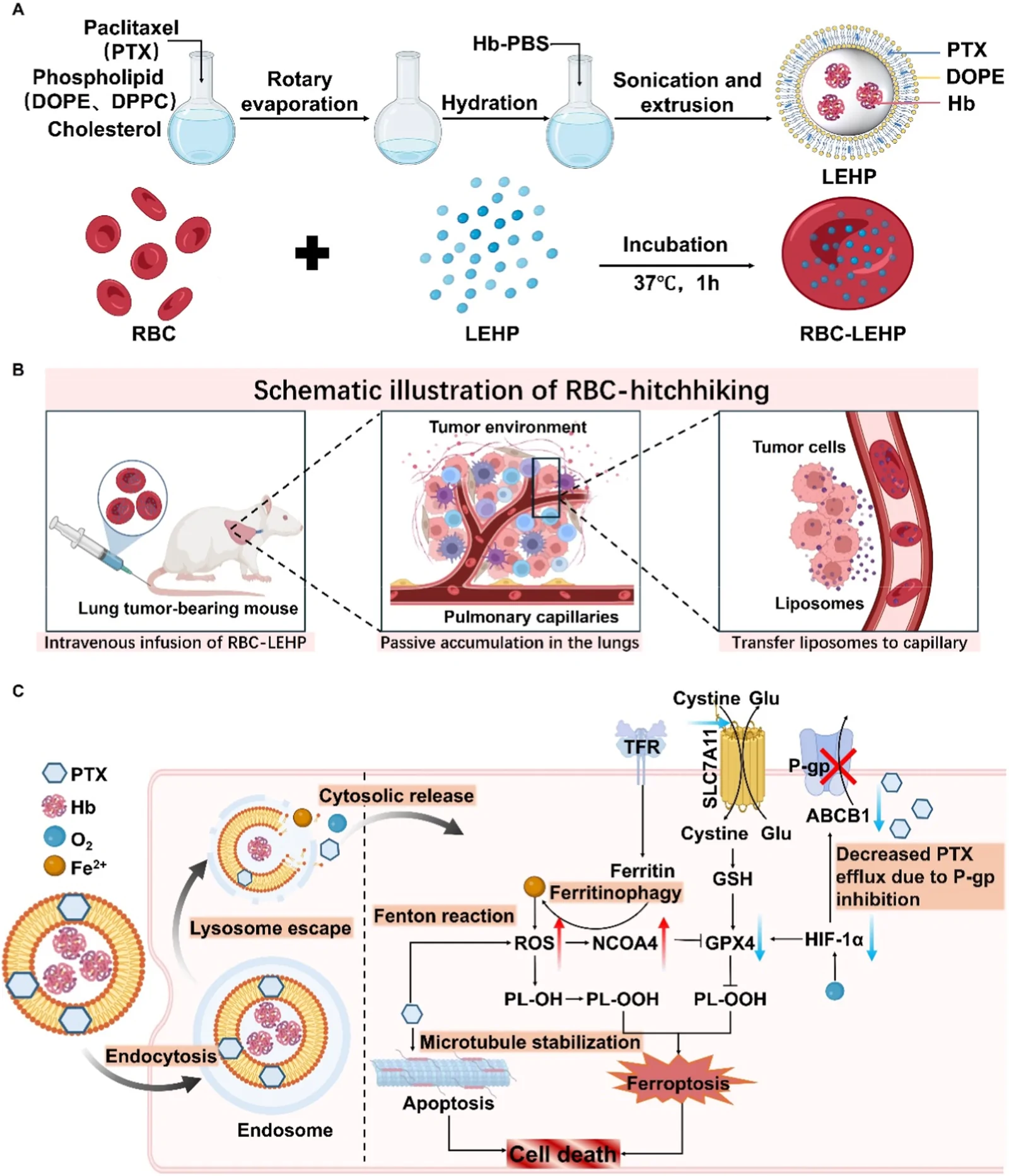
**Schematic representation of the RBC-hitchhiking-liposome drug delivery system (RBC-LEHP) for the TNBC lung metastasis chemotherapy.** (A) Fabrication of Liposomes/RBC-hitchhiking hybrid DDS (RBC-LEHP): LEHP liposomes were prepared by the thin-film hydration method and then incubated with red blood cells to obtain RBC-LEHP. (B) Pulmonary targeting mechanism: RBC-LEHP travels through the bloodstream and first reaches the pulmonary capillaries after tail-vein injection into BALB/c mice. Under physiological shear stress, the liposomes detach from the RBC carrier, resulting in localized accumulation in the lung capillaries. (C) Intracellular mechanism and synergistic antitumor efficacy: Following DOPE-mediated endosomal escape, the internalized LEHP releases haemoglobin to alleviate intratumoral hypoxia. This leads to the downregulation of the HIF-1α/P-gp resistance axis. Simultaneously, Hb-derived Fe^2+^ triggers a Fenton reaction, generating a ROS burst that drives lipid peroxidation and ferroptosis. This multi-modal cascade sensitizes TNBC cells to PTX, effectively reversing chemoresistance and suppressing tumour progression. (For interpretation of the references to colour in this figure legend, the reader is referred to the Web version of this article.)

## 2 Materials and methods

### 2.1 Materials

1,2-dipalmitoyl-sn-glycero-3-phosphocholine (DPPC) and 1,2-dioleoyl-sn-glycero -3-phosphoethanolamine (DOPE) were obtained from AVT Pharmaceutical Tech Co. Ltd. (Shanghai), and cholesterol was obtained from Aladdin. Paclitaxel (PTX) was purchased from Sigma Aldrich (St. Louis, MO, USA). For drug encapsulation and cellular uptake studies, liposomes were labeled with a green, fluorescent FITC-labeled phospholipid (Sigma-Aldrich) embedded in the lipid bilayer. For *in vivo* imaging, liposomes were labeled with the near-infrared fluorescent dye DiI (1,1′-dioctadecyl-3,3,3′,3′-tetramethylindocarbocyanine perchlorate, Thermo Fisher Scientific, Waltham, MA, USA). To label lysosomes, cells were stained with LysoTracker™ Red DND-99 (Thermo Fisher Scientific). For normalization of lysosomal fluorescence signal to cell number, Cell Tracker™ Green CMFDA (Thermo Fisher Scientific) was used as described. Cell viability was assessed using the MTT assay (3-(4,5-dimethylthiazol-2-yl)-2,5-diphenyltetrazolium bromide, Sigma Aldrich). Intracellular ferrous iron (Fe^2+^) was detected using the FerroOrange probe (Dojindo Molecular Technologies, Kumamoto, Japan), a novel fluorescent probe that enables live-cell imaging of Fe^2+^ with high sensitivity and specificity. FerroOrange working solution (1 μmol/L) was prepared in HBSS immediately before staining according to the manufacturer's protocol. Lipid peroxidation levels were assessed using the C11-BODIPY 581/591 lipid peroxidation sensor (Aladdin, Shanghai, China), a ratiometric fluorescent probe that shifts its emission peak upon oxidation and has been widely used for fluorescence imaging and flow cytometry analysis of ferroptosis. Mitochondrial membrane potential (ΔΨm) was measured using the JC-1 mitochondrial membrane potential assay kit (Aladdin, Shanghai, China), which uses the cationic dye JC-1 that accumulates in energized mitochondria in a potential-dependent manner (red aggregates at high ΔΨm and green monomers at low ΔΨm).

### 2.2 Preparation of hemoglobin and RBCs

Human blood was obtained from Chengdu or Deyang Blood Center. Venous blood was collected from healthy adults with informed consent. In total, 400 mL of whole blood was anticoagulated with 56 mL citrate–phosphate–dextrose. The leukocytes were removed by the leukocyte filter (Tianhe Pharmaceutical Company). Whole blood was centrifuged (3500 g, 5 min°C) to achieve red blood cells(RBCs). RBCs were stored with addition of red blood cell preservation solution and were washed 3 times (3500 g, 5 min, 4°C) with cold PBS before use. The hemoglobin was extracted by the hypotonic method with the use of cold double-distilled water at a ratio of 1:9. The hemoglobin supernatant solution was obtained after centrifugation (10000g, 5min, 4°C).

### 2.3 Preparation of LEP and LEHP

Liposomes were prepared by the conventional thin-film method. The lipids of LEP (liposomes encapsulate paclitaxel) were composed of DPPC, DOPE, and cholesterol with a molar ratio of 70:20:10, resulting in a total molar amount of 36 mM. PTX (3 mg) was dissolved in the above lipid chloroform solution. The lipid thin film was prepared by the rotary evaporator at 48°C for 1 h, and then it was hydrated with PBS for 30 min at 55°C, and then extruded through 0.2 μm and 0.1 μm polycarbonate membranes, respectively. The preparation of LEHP(liposomes encapsulating hemoglobin and paclitaxel)was the same as the LEP, except for the hydration step. The lipid thin film was hydrated with Hb-PBS(3 mg/mL)for 30 min at room temperature in a vacuum container. After hydration, the multilamellar liposomes (MLVs) were centrifuged (10000g, 4°C) to remove unencapsulated hemoglobin, and then MLVs were resuspended in PBS and extruded through 1.0 μm, 0.8 μm, 0.4 μm and 0.2 μm polycarbonate membranes, respectively. The size of these liposomes was verified by Delsa Nano C Particle Analyzer (Beckman Coulter, Inc) based on the dynamic light scattering (DLS) method. FITC-lipid incorporated liposomes (FITC-LEP and FITC-LEHP) were prepared by adding 0.5% mol of 1,2-dioleoyl-sn-glycerol-3-phosphoethanolamine-N-(carboxyfluorescein) (ammonium salt) (Avanti, USA) in the lipid solution.

### 2.4 Drug encapsulation efficiency

The encapsulation efficiency of PTX was determined by a reverse-phase high-performance liquid chromatography system (HPLC, Agilent Technologies, USA) using an SB-C18 column (Agilent, 20RBSX, 5 μm, 4.6 × 150 mm). The mobile phase is composed of 39% of double-distilled water, 37% of acetonitrile, and 24% of methanol with a flow rate of 0.8 mL/min. To measure the PTX in liposomes and RBC-lipo, 100 μL of each sample was mixed with 400 μL acetonitrile, vortexed for 2 min, and then centrifuged with 10,000 g for 2 min. The supernatant was filtered through a 0.22-μm filter, and the drug-encapsulated amount was measured by HPLC. Equation (1) was used to calculate encapsulation efficiency (EE %):

(1) EE(%)=AmountofPTXencapsulated/TotalamountofPTX×100%

Hemoglobin content was measured using a free hemoglobin assay kit (Beijing Ruierda company). For this purpose, 100 μL of sample was mixed with 1 μL of Triton X-100, vortexed for 2 min, and the hemoglobin concentration was then determined. The blood oxygen equilibrium curves of LEHP were measured by the HEMOX ANALYZER (TCS Scientific's Hemox Analyzer).

### 2.5 Preparation of RBC-LEP and RBC-LEHP

RBCs (2–3×10^8^) washed by PBS were incubated in the presence of liposomes (1–3 μM lipids) for 1h at 37°C. The suspension was gently rotated (30 rpm) during incubation. RBC hitchhiking LEP (RBC-LEP) or RBC hitchhiking LEHP (RBC-LEHP) was prepared after centrifugation (3500 g, 10 min, 4°C) and resuspended in PBS, and then RBC-LEP or RBC-LEHP was used immediately after preparation.

### 2.6 Cell culture

Lewis lung carcinoma (LLC) cells (FuHeng Biology, Shanghai) were cultured in DMEM (HyClone), and 4T1s (FuHeng Biology, Shanghai) were cultured in 1640 (HyClone), containing 10% fetal bovine serum (FBS, Transgen Biotech) and 1% penicillin–streptomycin. Cells were maintained at 37°C in a humidified atmosphere with 5% CO_2_. Adherent cells were passaged with 0.25% trypsin-EDTA when they reached 80-90% confluency.

### 2.7 Cell-based assays (HIF-1α, ROS, and cellular colocalization)

4T1 or LLC cells in the logarithmic growth phase were seeded at a density of 1 × 10^5^ cells per well in 6-well plates and cultured overnight under hypoxia (37°C, 1% O_2_, 5% CO_2_, and 94% N_2_) or normoxia (37°C, 20% O_2_, 5% CO_2_) conditions. Cells were then treated with basal medium containing LEP or LEHP (5 μg/mL PTX equivalent) for the indicated durations. After treatment, the drug-containing medium was removed, and cells were washed three times with PBS before subsequent processing. For HIF-1α production measurement, cells treated for 12 h were sequentially incubated with primary and secondary antibodies, washed with PBS, digested, and analyzed using imaging flow cytometry. For ROS production measurement, cells treated for 12 h or 24 h were collected, resuspended in PBS containing 10 μM DCFH-DA, and incubated at 37°C for 20 min. Fluorescence signals were measured by imaging flow cytometry at 485/538 nm (excitation/emission). For the assessment of lysosome and cell colocalization, FITC-labeled liposomes (FITC-LEP and FITC-LEHP) were prepared. Cells treated with the liposome formulations for 1, 2, 4 or 8 h were incubated with LysoTracker Red (50 nM) for 20 min, then centrifuged (300g, 3 min), resuspended, and analyzed by imaging flow cytometry.

### 2.8 Western blotting

Cells were lysed in RIPA buffer (50 mM Tris-HCl, pH 7.4, 150 mM NaCl, 1% NP-40, 0.5% sodium deoxycholate, 0.1% SDS) supplemented with protease and phosphatase inhibitor cocktail (Roche). Protein concentrations were determined using the BCA assay (Thermo Fisher Scientific). Equal amounts of protein (20–30 μg per lane) were separated by 10–12% SDS-PAGE and transferred onto PVDF membranes (Millipore). Membranes were blocked with 5% non-fat milk in Tris-buffered saline containing 0.1% Tween-20 (TBST) for 1 h at room temperature, followed by overnight incubation at 4°C with primary antibodies against HIF-1α (1:1000, Abcam), GPX4 (1:1000, Cell Signaling Technology), NCOA4 (1:800, Santa Cruz Biotechnology), P-gp (ABCB1, 1:500, Proteintech), and β-tubulin (1:5000, Abcam) as a loading control. After washing with TBST, membranes were incubated with HRP-conjugated secondary antibodies (1:5000, Cell Signaling Technology) for 1 h at room temperature. Protein bands were visualized using enhanced chemiluminescence (ECL) substrate (Bio-Rad) and imaged with a ChemiDoc MP system (Bio-Rad). Band intensities were quantified using ImageJ software (NIH), and relative protein expression levels were normalized to β-tubulin.

### 2.9 MTT

The MTT method was used to detect the cytotoxicity of drugs under hypoxia (37°C, 1% O_2_, 5% CO_2_, and 94% N_2_) or normoxia(37°C, 20% O_2_, 5% CO_2_) conditions. 4T1 or LLC in the logarithmic growth phase were seeded into 96-well plates at the density of 5000 per well and cultured overnight in hypoxic or normoxic incubators. After incubating different concentrations of LEP or LEHP under hypoxic or normoxic conditions for 48 h, MTT experiments were done according to the manufacturer's protocol. Calculation was according to equation II: cell viability (CV) = absorbance value (OD1) of sample group/absorbance value (OD0) of control group × 100%.

### 2.10 Hemolysis

When the incubation of RBCs with liposomes (LEP and LEHP) was completed, the solution was centrifuged at 3000 g, and the supernatant was used to detect the concentration of Hb, proceeding as described as the plasma-free hemoglobin assay kit (Beijing REAL-TECH).

### 2.11 Transmission electron microscopy (TEM)

Approximately 20 μL of the liposome sample was dropped onto a Parafilm sheet. A copper grid coated with a supporting film was placed onto the droplet with the film side facing down and incubated for 3 min at room temperature. The grid was then negatively stained with 2% phosphotungstic acid (PTA) for 1–2 min. After removal of excess staining solution with filter paper, the grid was air-dried at room temperature. Images were acquired using a JEM-1400FLASH transmission electron microscope (JEOL, Japan). The morphology of the liposomes was initially examined under low magnification to identify representative regions, followed by high-magnification imaging.

### 2.12 Characterization of RBCs

Osmotic fragility was assessed using a series of diluted sodium chloride solutions with concentrations ranging from 0.1% to 0.9%. Briefly, 50 μL of each untreated RBCs and liposomes combined RBCs were added to 1 mL of each diluted sodium chloride solution and incubated at room temperature for 30 min, followed by centrifugation at 3500*g* for 10 min. The supernatants were collected, and hemolysis was measured by a microplate reader (Bio-Tek Cytation 3) at a wavelength of 540 nm. To calculate the concentration that produced 50% hemolysis, the percent of hemolysis in each sodium chloride solution was plotted against the sodium chloride solution concentration.

RBC deformability was measured using a laser-diffraction Ektacytometer (LBY-BX, Beijing Precil Instrument Co., Ltd, China). RBCs were diluted 1:100 in a polyvinyl pyrrolidone solution (0.15 g/mL) and subjected to increasing shear stress at 37°C. The shear rate was increased by a faster rotation of the outer cup to cause RBCs deformation, and erythrocytes deformability (Elongation index, EI) was detected at four different shearing forces (50, 100, 200, 1000, and 2000 Pa) according to the laser diffraction principle.

Phosphatidylserine (PS) exposure and CD47 expression on the RBC surface were measured according to the protocols of the assay kits. Briefly, 2×107 RBCs or RBC-lipo were suspended in PBS and incubated with 5 μL of Annexin V-FITC (BD PharmingenTM) or CD47 (BD PharmingenTM) for 15 min in the dark and then detected by flow cytometry (Cytoflex, Beckman Coulter, Inc.).

### 2.13 In-vivo animal study

The 1 × 10^6^ 4T1-luciferase cells (4T1-Luc, Shanghai Fuheng Biocompany) were cultured in DMEM with 10% FBS. After the cells were cultured to 80-90%, washed with PBS, trypsinized, washed, and resuspended in PBS, they were then injected into BALB/C mice via the tail vein. The *in vivo* imaging system (IVIS) (PerkinElmer, Caliper IVIS Lumina LT S3) was used to determine the bioluminescence in the lungs of mice on day 5. After the establishment of the lung metastasis model, mice were randomized into four groups: LEP, LEHP, RBC-LEP, and RBC-LEHP. These groups received an equivalent 5 mg/kg PTX dose three times a week, a total of four times of administrations.

Liposome's accumulation in the organ was measured ex vivo using IVIS. DiI-stained liposomes were prepared according to the protocol of DiI dye kit (Beyotime, China). The samples were injected into BALB/C mice through the tail vein (PTX amount fixed at 5 mg/kg), and then the mice were sacrificed at different time points. The corresponding organs and blood samples were dissected and imaged by IVIS. An excitation wavelength of 549 nm and an emission wavelength of 565 nm were used. The total radiant efficiency (p/s/cm2/sr/μW/cm^2^) was determined using Living Image software from IVIS (PerkinElmer).

### 2.14 Ethics statement

All animal experiments were approved by the Institutional Animal Care and Use Committee (IACUC) of the Institute of Blood Transfusion, Chinese Academy of Medical Sciences (approval No. 2023024). All methods were carried out in accordance with the relevant guidelines and regulations of the national standard “Laboratory animal—Guideline for ethical review of animal welfare" (GB/T 35892-2018).

### 2.15 Immunohistochemistry

Tissues were fixed in 10% formaldehyde, dehydrated, embedded, and sectioned. Sections were deparaffinized, rehydrated, and treated with 3% H_2_O_2_ for 25 min at room temperature in the dark. After blocking with 5% normal goat serum for 30 min at room temperature, sections were incubated with primary antibody overnight at 4°C, followed by horseradish peroxidase-conjugated secondary antibody for 50 min at room temperature. Signals were visualized using DAB substrate, and sections were counterstained with hematoxylin, differentiated, dehydrated, and mounted. Images were acquired using a digital slide scanner, and integrated optical density was analyzed using Image-Pro Plus.

### 2.16 DIA-based quantitative proteomics

Mobile phase A (100% water, 0.1% formic acid) and mobile phase B (80% acetonitrile, 0.1% formic acid) were prepared. For each sample, 4 μg of supernatant was mixed with 0.8 μL of iRT reagent and loaded onto the analytical system. The peptides were separated on an EASY-nLC™ 1200 UHPLC system equipped with a home-packed trap column (4.5 cm × 75 μm, 3 μm) and a home-packed analytical column (15 cm × 150 μm, 1.9 μm). Mass spectrometry analysis was performed on a Q Exactive™ HF-X mass spectrometer coupled with a Nanospray Flex™ (ESI) ion source. The spray voltage was set to 2.1 kV, and the ion transfer tube temperature was maintained at 320°C. Data-independent acquisition (DIA) mode was employed with a full-scan range of *m*/*z* 350–1500. Full-MS spectra were acquired at a resolution of 60,000 (at *m*/*z* 200), with a C-trap maximum fill time of 20 ms and an automatic gain control (AGC) target of 5 × 10^5^. For MS/MS, higher-energy collisional dissociation (HCD) was used at a normalized collision energy of 27%, and the fragment spectra were acquired at a resolution of 30,000 (at *m*/*z* 200) with an AGC target of 1 × 10^6^. Raw data files (raw) were generated for subsequent analysis.

### 2.17 Statistical analysis

All statistical analyses were performed using SPSS 7.0. All data were expressed as the mean ± standard deviation. For normally distributed data, the significance of mean differences was determined using unpaired Student's t-test between two groups or ANOVA followed by Newman–Keuls multiple comparison test among multiple groups. A difference was considered statistically significant if the P-value was <0.05.

## 3 Results and discussion

### 3.1 Characterization of liposomes and RBCs-liposomes

Physicochemical characterization (Table S1) revealed that LEP and LEHP formulations exhibited mean diameters between 100 and 200 nm, with polydispersity index (PDI) of < 0.2, indicating a highly homogeneous population. Both formulations maintained a slight negative zeta potential. The encapsulation efficiency (EE) of paclitaxel was 83.32% ± 4.43% for LEP and 58.27% ± 2.55% for LEHP; the reduced EE in LEHP may be attributed to the incorporation of hemoglobin into the aqueous phase of the liposomes.

We characterized the oxygen affinity of the LEHP formulation by determining its P50 value—the partial pressure of oxygen at which hemoglobin is 50% saturated, a key biophysical parameter governing oxygen delivery efficiency. The P50 measurement data are presented in Supplementary Fig. S2. The P50 of LEHP was measured to be approximately 10.20 mmHg, substantially lower than that of normal adult hemoglobin (∼26.5 mmHg under physiological conditions of 37°C and pH 7.4). This reduced P50 reflects a left-shifted oxygen dissociation curve, indicative of increased oxygen affinity. LEHP thus exhibits enhanced oxygen loading in the pulmonary circulation while maintaining a thermodynamic gradient favoring oxygen release under the low pO_2_ conditions characteristic of the tumor microenvironment.

The morphology of the liposomes was further examined by transmission electron microscopy (TEM). As shown in Fig. 2C (LEP) and 2D (LEHP), both formulations exhibited well-defined spherical structures with smooth surfaces and uniform size distribution, which is consistent with the DLS measurements. No substantial morphological differences were detected between LEP and LEHP, and our observations indicate that hemoglobin incorporation did not compromise the structural integrity of the liposomes.

**Fig. 2:**
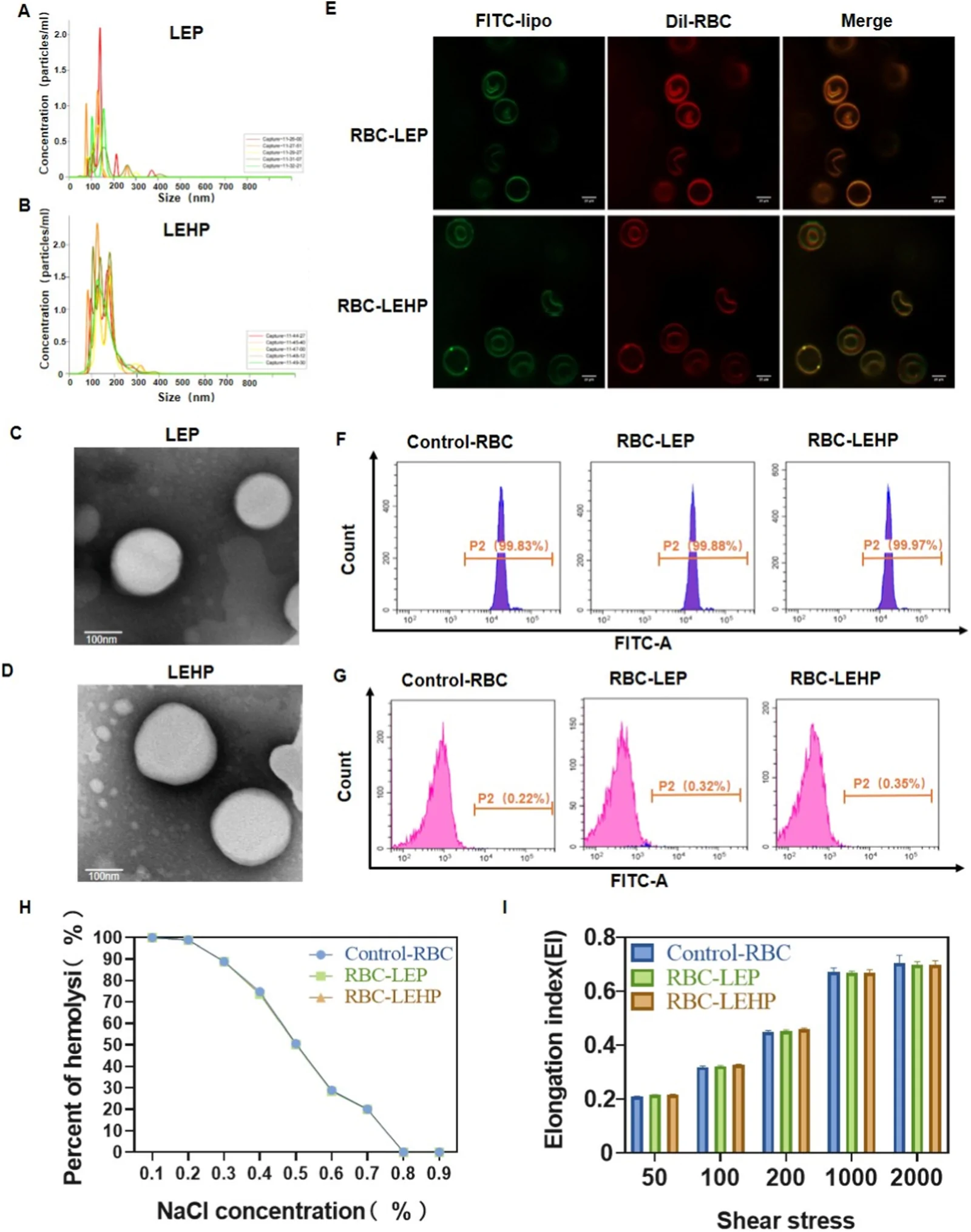
(A) Particle size distribution of LEP and (B) LEHP. (C) TEM image of LEP and (D) LEHP. (E) Confocal microscopy images of RBCs after liposome incubation. Green, FITC-labeled liposomes; red, DiI-labeled RBCs. Scale bar:20 μm. (F) CD47 expression on control RBCs and RBC-lipo. (G) Phosphatidylserine (PS) exposure on control RBCs and RBC-lipo. (H) Deformability of RBC-lipo and control RBCs. n = 5 independent measurements. (I) Osmotic fragility of RBC-lipo and control RBCs. n = 5. (For interpretation of the references to colour in this figure legend, the reader is referred to the Web version of this article.)

Optimization of the RBC-hitchhiking system (Table S2) identified a 3 μM liposomes to 3 × 10^8^ RBCs ratio as the ideal incubation parameter for maximal binding. Confocal microscopy (Fig. 2E) confirmed that liposomes (FITC, green) uniformly coated the RBC membrane (Dil, red) while preserving the native biconcave disc morphology.

Critically, the biomimetic integrity of the RBCs remained intact post-incubation. CD47 expression level were maintained (Fig. 2F), ensuring preservation of the immune evasion function (“don't eat me” signaling) via the CD47–SIRPα axis [17]. Furthermore, there was no significant increase in phosphatidylserine (PS) exposure observed via Annexin V staining (Fig. 2G), and osmotic fragility profiles remained consistent with native RBCs (Fig. 2H). In term of hemodynamic, the deformation index (EI) was not significantly altered (Fig. 2I), indicating that the RBC-LEHP complex retains the essential mechanical flexibility required to traverse splenic sinuses and pulmonary capillaries.

In summary, the RBC-liposome system achieves high-density drug loading while maintaining the intrinsic biological and mechanical properties of the erythrocyte carrier, providing a robust foundation for targeted delivery.

### 3.2 Intracellular hypoxia alleviation and synergistic cytotoxicity

Tumor hypoxia stabilizes HIF-1α, driving metabolic reprogramming and multidrug resistance [18,19]. Our result (Fig. 3A–B, H-I) confirmed that LEHP treatment significantly downregulates HIF-1α expressntion in both LLC and 4T1 cells compared to the hemoglobin-free LEP control in both normoxic (20% O_2_) and hypoxic (5% O_2_) conditions. Notably, 4T1 cells exhibited higher baseline HIF-1α levels, reflecting a more severe hypoxic state characteristic of aggressive, metastatic phenotypes. Beyond mere oxygenation, LEHP facilitates a robust intracellular ROS burst (Fig. 3C–D, J-K). This dual-action – alleviating hypoxia while simultaneously elevating ROS generation – suggests that Hb acts as a therapeutic “switch”, shifting the tumor cell from a survival-oriented hypoxic state to a pro-apoptotic oxidative state.

**Fig. 3:**
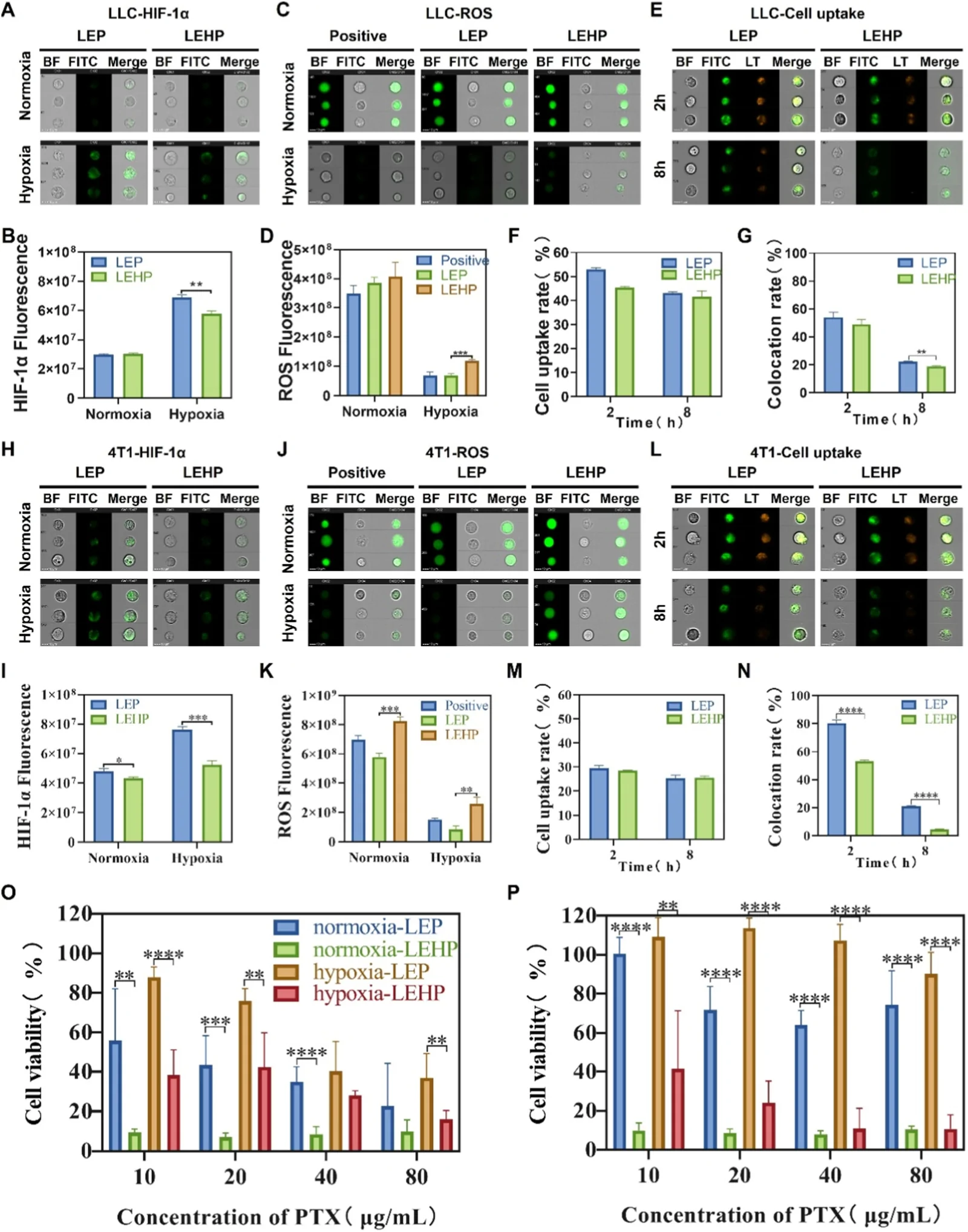
**Evaluation of cellular uptake, hypoxia alleviation, and synergistic cytotoxicity in LLC and 4T1 cells.** (A-D, H-K) Hypoxia alleviation and ROS generation: representative immunofluorescence images and corresponding quantification of (A, B, H, I) HIF-1α expression and (C, D, J, K) intracellular ROS production in LLC and 4T1 cells following 12h incubation under normoxic or hypoxic conditions. (E-G, L-N) Cellular internalization and lysosomal escape: (E, L) Representative imaging flow cytometry showing colocalization of FITC-labeled liposomes (green) and LysoTracker-labeled lysosomes (red). Corresponding quantification of (F, M) total liposomal uptake and (G, N) colocation rate for liposome-lysosomal colocalization. (O, P) Relative cell viability of (O) LLC and (P) 4T1 cells after 48h treatment with varying liposomal formulations. Data are presented as mean ± SD (n = 3 for imaging/flow; n = 5 for viability). Scale bars: 10 μm (IF) and 7 μm (Flow). *P < 0.05, P < 0.01, ***P < 0.001, ****P < 0.0001. (For interpretation of the references to colour in this figure legend, the reader is referred to the Web version of this article.)

Efficient intracellular delivery is often throttled by lysosomal sequestration. While both formulations utilize the pH-sensitive phospholipid DOPE for endosomal escape via the “proton sponge” effect, LHP exhibited significantly accelerated escape kinetics as early as 2 h post-incubation (Fig. 3E–G, L-N). We propose a ROS-dependent positive feedback mechanism for this enhanced escape [20]:

1. Initial Release: The acidic lysosomal environment triggers DOPE-mediated membrane destabilization, releasing encapsulated Hb.
2. Fenton Chemistry: Liberated Fe^2+^ from Hb reacts with local peroxides to generates highly reactive hydroxyl radicals (·*OH*).
3. Peroxidative destabilization: These ROS directly attack the polyunsaturated fatty acids (PUFAs) concentrated in the lysosomal membrane.
4. Amplified escape: Lipid peroxidation increases membrane permeability, accelerating the cytosolic release of both Hb and PTX.

Cytotoxicity assays (Fig. 3O and P) revealed that while 4T1 cells were largely refractory to PTX alone (LEP), LEHP possessed stronger cytotoxicity in a concentration-dependent manner. This reversal is particularly pronounced in 4T1 cells due to their adaptation to chronic hypoxia (marked by high LDH-A expression) [21] [22].

The superior efficacy of LEHP can be attributed to “re-oxygenation injury". In chronically hypoxic cells, the sudden influx of Hb-delivered oxygen overwhelms the cell's adapted antioxidant defences. This oxygen surge, combined with the ROS generated via the Fenton reaction, triggers a catastrophic oxidative collapse [23]. Consequently, the RBC-LEHP system effectively bypasses the metabolic barriers of TNBC, transforming a survival advantage (hypoxia) into a therapeutic vulnerability.

### 3.3 Mechanistic dissection of LEHP-induced ferroptosis and resistance reversal

To dissect the mechanistic basis underlying LEHP-enhanced cytotoxicity under hypoxia, we performed MTT rescue experiments using the specific ferroptosis inhibitor Fer-1. As shown in Fig. 4A, Fer-1 co-treatment profoundly reversed LEHP-induced cell death across all tested PTX concentrations, unequivocally establishing Hb-mediated ferroptosis as the primary route of enhanced cytotoxicity. Notably, at the highest PTX concentration, the LEHP + Fer-1 group retained a modest viability reduction, suggesting a minor ferroptosis-independent component—most plausibly enhanced apoptosis resulting from hypoxia relief that sensitizes cells to PTX. Thus, LEHP engages a dual-pronged death machinery, with ferroptosis as the dominant modality and apoptosis as a complementary route under high-drug-load conditions. By contrast, Fer-1 exerted negligible rescue on LEP-treated cells, confirming that PTX alone under hypoxia proceeds through apoptosis with minimal ferroptotic contribution, consistent with the established paradigm that paclitaxel does not provoke ferroptosis in the absence of iron-loading stimuli.

**Fig. 4:**
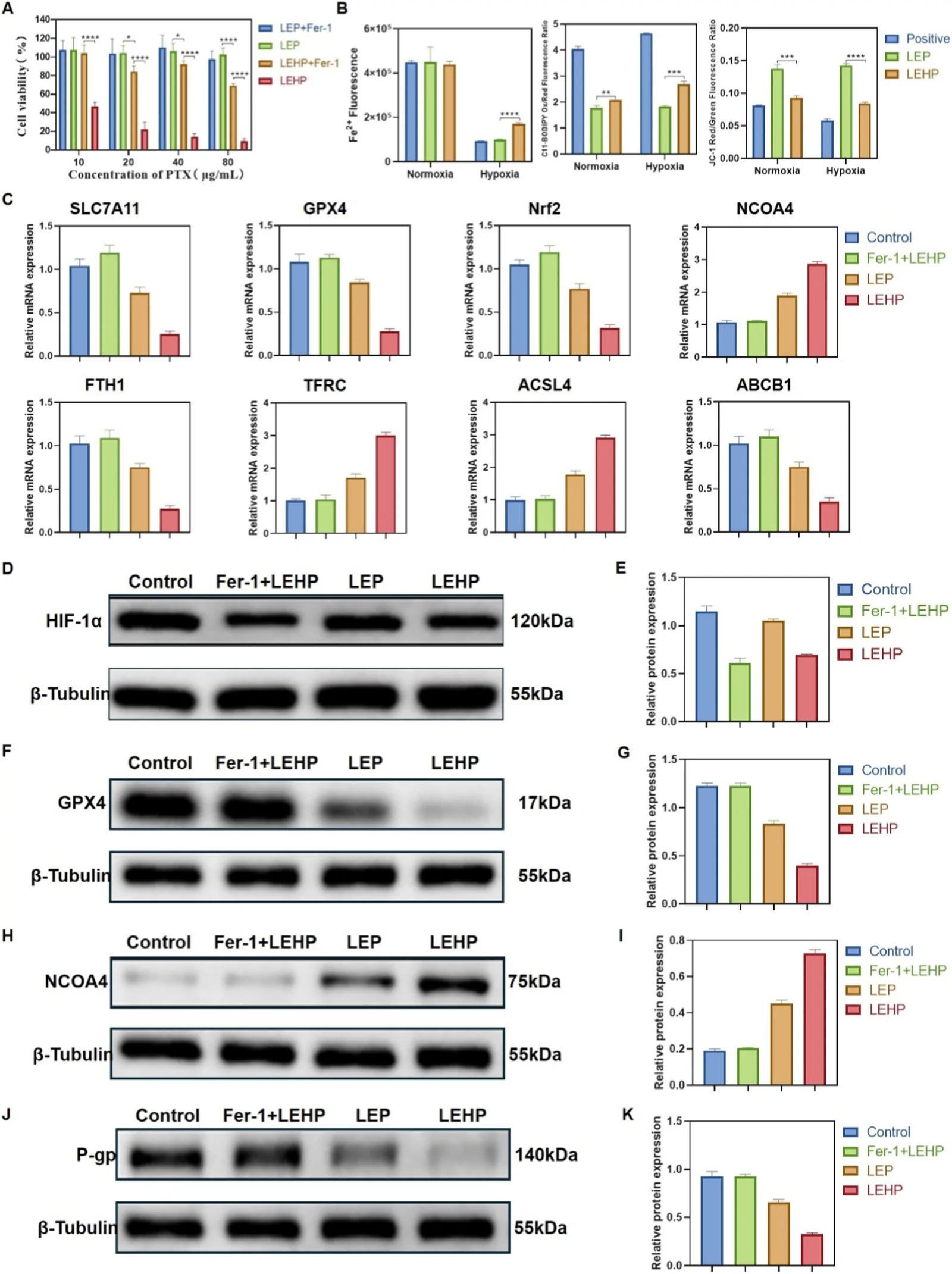
Mechanistic validation of LEHP-induced ferroptosis and resistance axis reversal in 4T1 cells. (A) Effects of ferroptosis inhibition on LEHP-induced cytotoxicity under hypoxic conditions. Cell viability was determined by MTT assay. Data are presented as mean ± SD (*n* = 6, *P < 0.05, P < 0.01, ***P < 0.001, ****P < 0.0001). (B) Quantitative imaging flow cytometry analysis of intracellular ferrous iron (Fe^2+^) accumulation via FerroOrange staining, lipid peroxidation via C11-BODIPY, and mitochondrial membrane potential shifts via JC-1 ratiometric fluorescence after 12h (iron) or 24h (lipid/JC-1) treatment under normoxic or hypoxia. (C) Real-time quantitative PCR (qPCR) profiling of ferroptosis- and resistance-associated genes in 4T1 cells under hypoxia following 24h treatments with indicated formulations ± ferrostatin-1 (Fer-1) pretreatment. (D, F, H, J) Representative Western blot bands and (E, G, I, K) corresponding densitometric quantification of (D, E) HIF-α, (F, G) GPX4, (H, I) NCOA4, and (J, K) P-gp expression levels under hypoxic conditions after 24h incubation. Data are normalized to internal loading controls (*β*-actin for qPCR; *β*-tubulin for Western blot) and presented as mean ± SD (*n* = 3; 5000 cells collected per flow cytometry experiment).

Having established ferroptosis as the predominant phenotypic driver, we next sought to validate its molecular execution and downstream impact on the chemoresistance machinery. Evaluation of the three hallmarks of ferroptosis (Fig. 4B) revealed that LEHP treatment markedly elevated intracellular labile ferrous iron (Fe^2+^) and accelerated lipid peroxide (LPO) accumulation compared to the LEP control. This concurrent iron overload and LPO surge drove a profound collapse of the mitochondrial membrane potential, serving as both a crucial executioner mechanism and a definitive indicator of ferroptosis induction.

To elucidate the molecular architecture driving this phenotypic shift, we quantified key ferroptosis- and resistance-related markers via qPCR and Western blot (Fig. 4C). To definitively confirm that the therapeutic efficacy of LEHP is driven by ferroptosis, cells were pretreated with the lipophilic radical scavenger ferrostatin-1 (Fer-1). LEHP significantly downregulated the transcription factor *Nrf2* and its downstream targets, *SLC7A11* (a key subunit of the cystine/glutamate antiporter) and *GPX4*. This coordinated suppression deactivates the glutathione (GSH)-dependent lipid peroxide clearance pathway, leaving cellular membranes vulnerable to oxidative attack.

Furthermore, LEHP treatment upregulated the transferrin receptor (*TFRC*) to increase iron influx, while simultaneously driving *NCOA4*-mediated ferritinophagy and downregulating the iron-storage protein *FTH1*. This dual action systematically degrades intracellular ferritin, liberating free Fe^2+^ into the labile iron pool to fuel the Fenton reaction. Concurrently, the key lipid-remodeling enzyme *ACSL4* was markedly upregulated, providing an abundance of polyunsaturated fatty acid (PUFA) substrates to accelerate membrane lipid peroxidation.

Crucially, LEHP treatment mediated a sharp downregulation of *ABCB1* (P-glycoprotein/P-gp), the primary ATP-dependent efflux pump responsible for PTX resistance. Linking back to the hypoxia alleviation shown in Fig. 3, the suppression of the HIF-1α transcription factor directly removes the transcriptional activation of *ABCB1*. This establishes that LEHP reverses chemoresistance through a direct **hypoxia - > HIF-1α - > P-gp** suppression axis, effectively sensitizing the cells to PTX-mediated cytotoxicity.

Collectively, these data demonstrate that under chronic hypoxia, HIF-1α acts as a transcriptional brake that suppresses ferroptosis by upregulating antioxidant pathways. By delivering oxygen and Fe^2+^ simultaneously, the LEHP system releases this brake, coordinates an intracellular iron/lipid storm, collapses mitochondrial integrity, and dismantles the P-gp efflux machinery to eradicate chemo-resistant TNBC cells.

### 3.4 In-vivo biodistribution and pulmonary accumulation

To evaluate the lung-targeting efficiency of the developed biomimetic platform, the biodistribution and organ accumulation profiles of liposomes (Lipo) and RBC-hitchhiking liposomes (RBC-Lipo) were monitored via IVIS imaging system in a 4T1 breast cancer lung metastasis mouse model.

Ex vivo organ imaging (Fig. 5A) demonstrated that the pulmonary fluorescence signal in the RBC-Lipo group peaked rapidly, sustained high intensity for 6 h, and remained detectable up to 48 h post-injection. This prolonged retention stands in stark contrast to conventional long-circulating strategies (e.g., PEGylation), which often fail to achieve sufficient target-site accumulation [24]. Quantification of the organ distribution (Fig. 5B and C) revealed that the RBC-hitchhiking strategy achieved a 2.7-fold increase in pulmonary fluorescence intensity, and a 4.6-fold enhance emnt in the lung-to-liver ratio compared to the liposome-alone group. These findings demonstrate that RBC hitchhiking effectively bypasses liver and spleen sequestration by the reticuloendothelial system (RES), yielding both rapid pulmonary enrichment and an extended therapeutic window tailored for metastatic TNBC therapy.

**Fig. 5:**
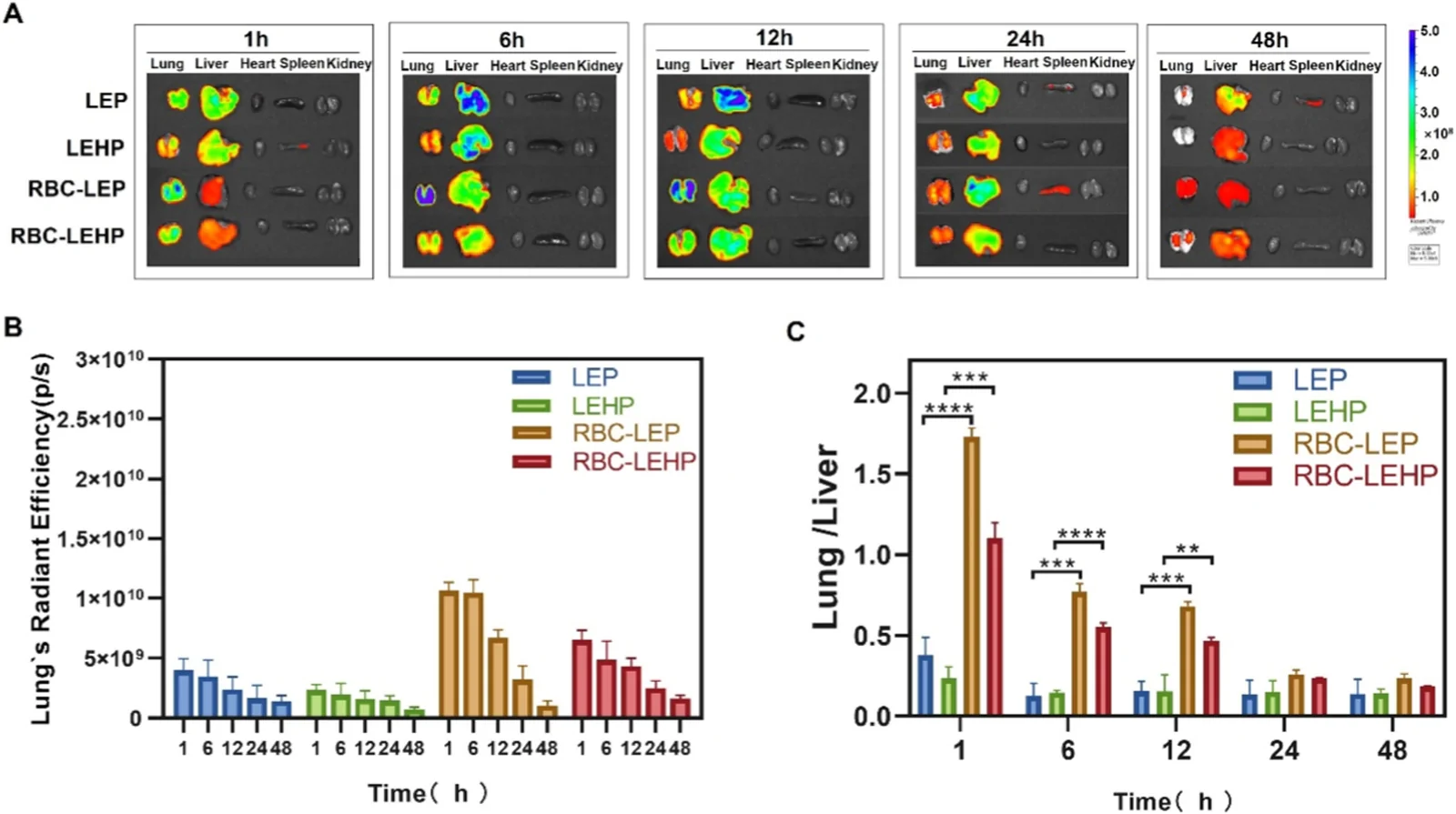
Organ distribution of liposomes in mice bearing 4T1 breast cancer lung metastases (n = 4). DiI-labeled liposomes were intravenously injected into BALB/c mice with 4T1 lung metastases (PTX: 5 mg/kg). Mice were euthanized at different time points (1, 6, 12, 24 and 48 h), and the heart, liver, spleen, lung and kidney were collected for ex vivo fluorescence imaging. (A) IVIS fluorescence images of liposomes in each group; (B) quantitative fluorescence intensity in the lungs; (C) lung-to-liver fluorescence intensity ratio.

It is noteworthy that the RBC-LEHP group exhibited detectable fluorescence signals in the liver at 24 h post-injection. This is consistent with the known hepatic clearance pathway of liposomal formulations and RBC-hitchhiked nanoparticles. Importantly, H&E staining of liver tissues (Supplementary Fig. 1) revealed no obvious histological abnormalities in the RBC-LEHP group compared with the control group, suggesting that the liver accumulation does not translate into overt hepatotoxicity. Nevertheless, future optimization of the formulation to reduce non-specific hepatic uptake would be valuable for further improving the safety profile.

### 3.5 Therapeutic efficacy of RBC-LEHP in a 4T1 lung metastasis model

The therapeutic efficacy of the RBC-LEHP was evaluated in a bioluminescence 4T1 TNBC lung metastasis mouse model (Fig. 6). Paclitaxel monotherapy (LEP; lacking hemoglobin and RBC-hitchhiking) failed to arrest tumor progression, confirming that conventional chemotherapy cannot overcome the multi-drug resistance endemic to the hypoxic 4T1 niche. To decouple the biochemical and pharmacokinetic mechanisms, we evaluated LEHP (Hb/PTX liposomes) and RBC-LEP (RBC-hitchhiking liposomes without Hb). The LEHP group exhibited enhanced tumor inhibition compared to LEP, validating that Hb-mediated re-oxygenation and Fe^2+^-driven ferroptosis sensitize resistant TNBC cells to PTX; however, its efficacy was limited by a reliance on passive tissue accumulation. Conversely, the RBC-LEP group achieved high initial entrapment within the pulmonary microvasculature via erythrocyte dynamics but failed to sustain tumor suppression. This demonstrates that augmenting local drug delivery without modulating the hypoxic microenvironment is insufficient to abrogate hypoxia-driven chemoresistance.

**Fig. 6:**
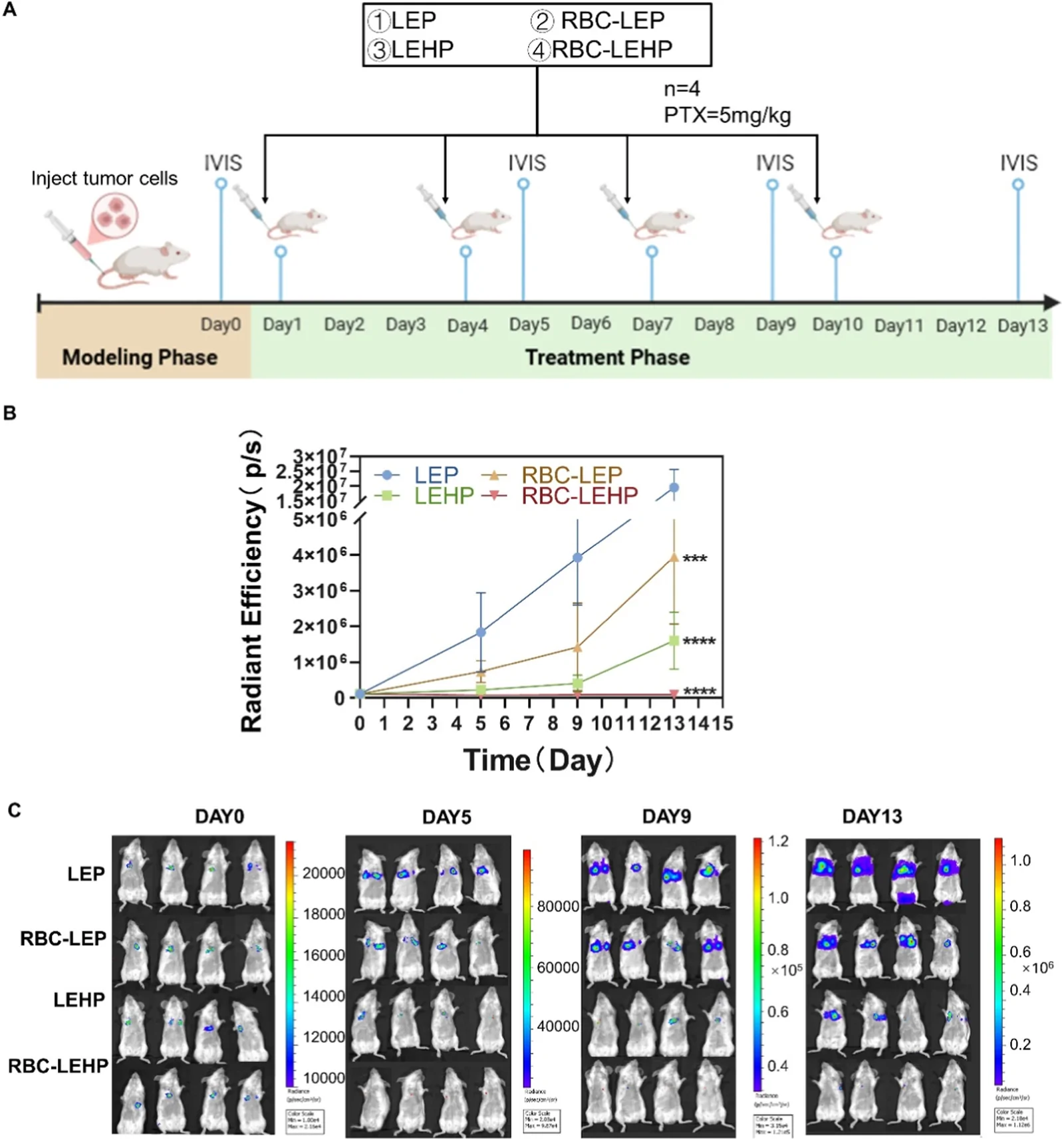
Therapeutic experiment in the 4T1 breast cancer lung metastasis model (n = 4). A 4T1 breast cancer metastasis model was established in BALB/c mice to evaluate therapeutic efficacy. On day 0, successfully engrafted mice were randomly assigned to four groups: LEP, LEHP, RBC-LEP, and RBC-LEHP. On day 1, each group received intravenous (tail vein) injections every two days for a total of four doses. Tumor bioluminescence signals were detected using an IVIS system on days 5, 9, and 13. On day 13, mice were euthanized, and the heart, liver, spleen, lung, and kidney were collected. (A) Schematic of the treatment regimen; (B) tumor growth curve based on *in vivo* bioluminescence signal intensity; (C) representative bioluminescence images on day 0, 5, 9, and 13.

The fully integrated RBC-LEHP platform achieved profound, sustained tumor regression, maintaining the lowest bioluminescence kinetics throughout the study. At the day 13 endpoint, the pulmonary bioluminescence signal of the RBC-LEHP cohort was (7.70 ± 2.86) × 10^4^, representing a three-orders-of-magnitude reduction compared to the LEP group (2.19 ± 0.37) × 10^7^. These data establish a dual-engine therapeutic paradigm: encapsulated hemoglobin serves as the biochemical switch to dismantle the hypoxic survival machinery and trigger ferroptosis, while RBC-hitchhiking acts as the pharmacokinetic driver that concentrates this therapeutic cascade within pulmonary metastatic lesions.

Histopathological analysis via H&E staining (Fig. 7A) revealed that paclitaxel monotherapy (LEP) resulted in severe parenchymal disruption, extensive alveolar necrosis, inflammatory infiltration, and massive metastatic nodules. While these pathological features were partially attenuated in the LEHP cohort, the fully integrated RBC-LEHP group maintained intact bronchial architectures, normal epithelial morphologies, and an absence of edema or fibrotic proliferation, confirming superior therapeutic efficacy. Furthermore, H&E profiling of major off-target organs (heart, liver, spleen, kidney) demonstrated no systemic toxicity, establishing the favorable biosafety profile of the RBC-LEHP platform.

**Fig. 7:**
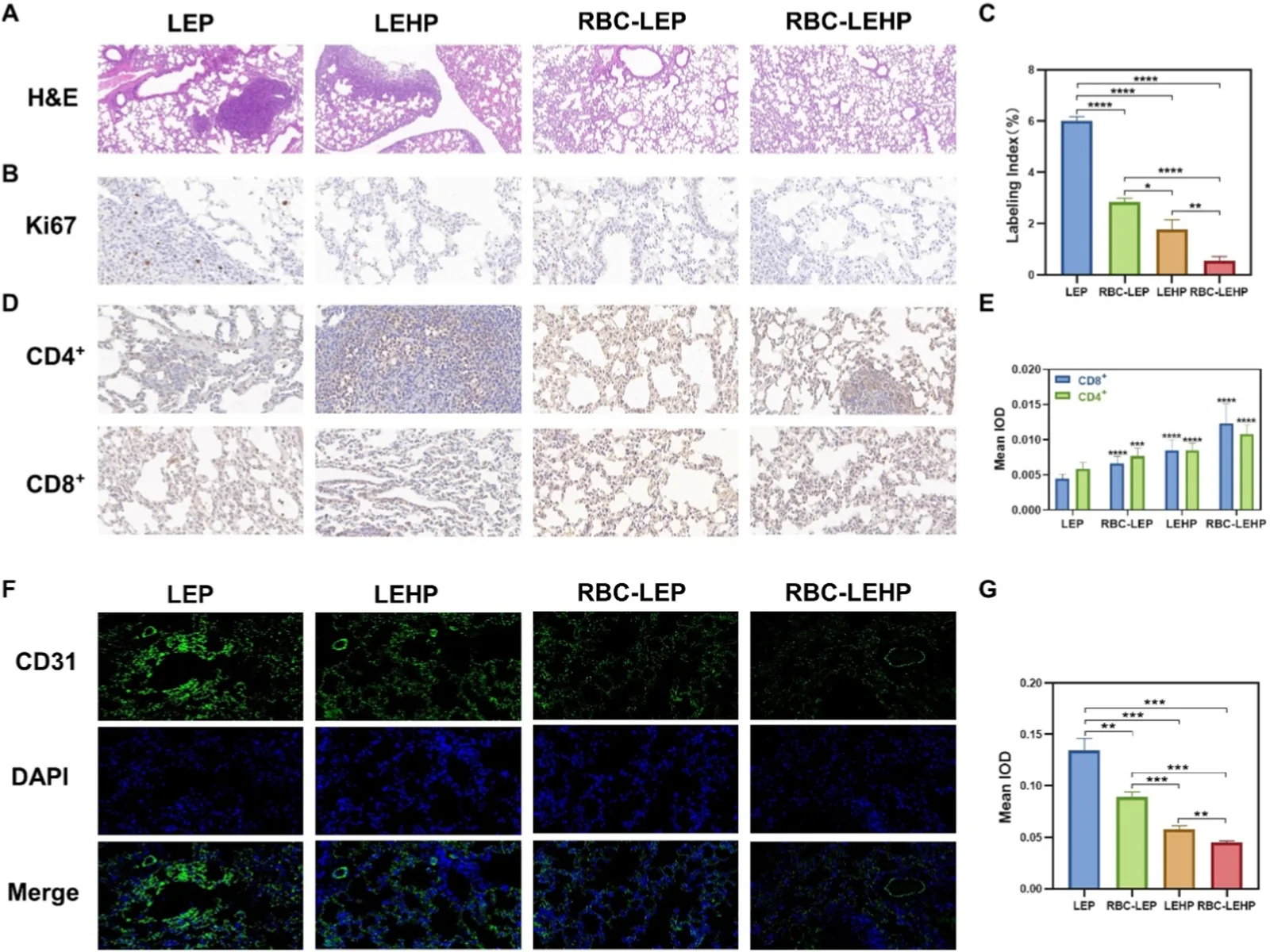
Related assays of lung tissues from mice with the 4T1 breast cancer lung metastasis model at the end of the therapeutic administration experiment (n = 4). (A) H&E staining of lung from each group; (B–C) representative images and quantification of Ki67 expression in lung tissues; (D–E) representative images and quantification of CD4^+^/CD8^+^ cell populations in lung tissues; (F–G) representative images and quantification of CD31 expression in lung tissues.

Immunohistochemical quantification confirmed that this structural preservation was driven by a concurrent suppression of tumour proliferation and angiogenesis. RBC-LEHP treatment significantly reduced the frequency of proliferating cells compared to the LEP group (Fig. 7B and C). Concurrently, analysis of the endothelial marker CD31 (Fig. 7F and G) showed a marked reduction in the density of tumor microvessels. This suppressed microvessel density (MVD) correlates with the downregulation of the hypoxia-driven angiogenic cascade downstream of Hb-mediated re-oxygenation.

Beyond direct cytotoxicity, the RBC-LEHP platform successfully remodelled the tumor immune microenvironment. Immunohistochemical (IHC) staining (Fig. 7D and E) demonstrated that RBC-LEHP treatment markedly elevated the infiltration of both CD4^+^ and CD8^+^ T cells, while significantly optimizing the CD8^+^/CD4^+^ ratio compared to the LEP control. This robust shift indicates a successful transition from an immunologically suppressed “cold" tumor to an active, inflamed “hot" tumor microenvironment. This study primarily focused on local immune modulation within the tumor microenvironment; however, systemic immune responses in peripheral lymphoid organs (e.g., spleen and lymph nodes) were not evaluated. Future investigations incorporating comprehensive immune profiling of these organs would provide a more complete picture of the host immune status and further deepen our mechanistic understanding. Collectively, these histopathological and immunophenotypic findings validate the multi-mechanistic superiority of the RBC-LEHP system in safely eradicating metastatic TNBC.

To validate the multi-mechanistic cascade of the RBC-LEHP platform *in vivo*, we examined key markers of hypoxia, oxidative stress, ferroptosis and metastasis in lung tissues (Fig. 8).

**Fig. 8:**
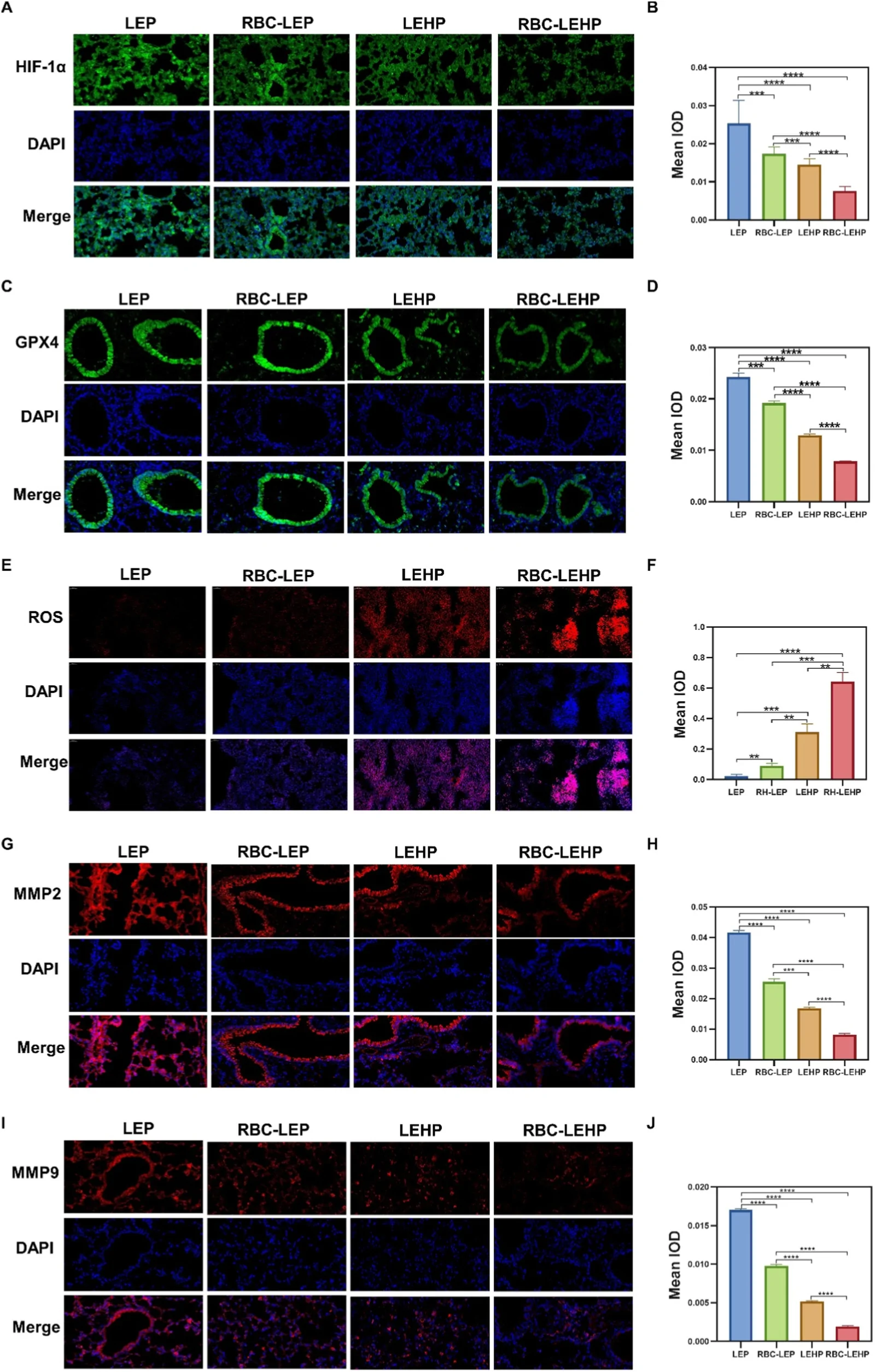
(A-B) Representative images and quantification of HIF-1α expression in lung tissues; (C-D) representative images and quantification of GPX4 levels in lung tissues; (E-F) representative images and quantification of ROS expression in lung tissues; (G-H) representative images and quantification of MMP2 expression in lung tissues; (I-J) representative images and quantification of MMP9 expression in lung tissues. n = 3.

Fig. 8A and B showed that HIF-1α expression was markedly elevated in the LEP group, indicating severe tumor hypoxia, whereas RBC-LEHP treatment significantly reduced HIF-1α levels, demonstrating that hemoglobin-mediated oxygen delivery effectively alleviated the hypoxic tumor microenvironment. Previous studies have shown that hypoxia suppresses ferroptosis through transcriptional upregulation of antioxidant genes (e.g., *SLC7A11*, *GPX4*) and stabilization of HIF-1α, which indirectly limits lipid peroxidation. Thus, by alleviating hypoxia, RBC-LEHP removes this natural barrier and creates permissive conditions for ferroptosis initiation.

Consistent with our *in vitro* findings, GPX4 expression was significantly downregulated in the RBC-LEHP treatment group (Fig. 8C and D). As GPX4 is the primary scavenger of lipid hydroperoxides, its downregulation indicates a catastrophic collapose of the tumor's antioxidant defense mechanism. This loss of protective capacity inversely correlated with HIF-1α downregulation, confirming that relieving tissue hypozia abolishes the cell's adaptive resistance to oxidative stress. Direct quantification of tissue ROS (Fig. 8E and F) demonstrated significantly elevated oxidative signals in the Hb-containing cohorts (LEHP and RBC-LEHP). This elevation arises from two complementary mechanisms: Fe^2+^ released from hemoglobin drives the Fenton reaction, converting H_2_O_2_ into highly cytotoxic hydroxyl radicals (·OH) that directly induce oxidative damage, while paclitaxel simultaneously amplifies baseline intracellular ROS production.

To assess the platform's impact on the metastatic machinery, we monitored the expression of matrix metalloproteinases MMP2 and MMP9 (Fig. 8G–J). The RBC-LEHP group exhibited a marked reduction in both markers compared to the LEP control, confirming that the combination of ferroptosis induction and chemo-sensitization effectively deactivates the enzymes responsible for extracellular matrix remodeling and tumor invasion.

These *in vivo* molecular insights confirm that the RBC-LEHP platform successfully coordinates passive pulmonary sequestration and active tumor internalization to drive a highly localized **hypoxia alleviation - > iron/ROS surge - > GPX4 inactivation** cascade, safely reversing chemoresistance and arresting metastatic progression in advanced TNBC.

### 3.6 Multi-mechanistic proteomic reprogramming of hypoxia, ferroptosis and drug resistance

To elucidate the antitumor molecular mechanisms of RBC-LEHP, we performed high-throughput proteomic analysis on the harvested lung tissues (Fig. 9). Principal component analysis and hierarchical clustering revealed distinct separation between the RBC-LEHP and the LEP control cohorts, indicating unique global protein expression reprogramming. KEGG enrichment analysis (Fig. 9A and B) further demonstrated that significant enrichment of these proteins in pathways governing ferroptosis, cell cycle regulation, and tumorigenesis.

**Fig. 9:**
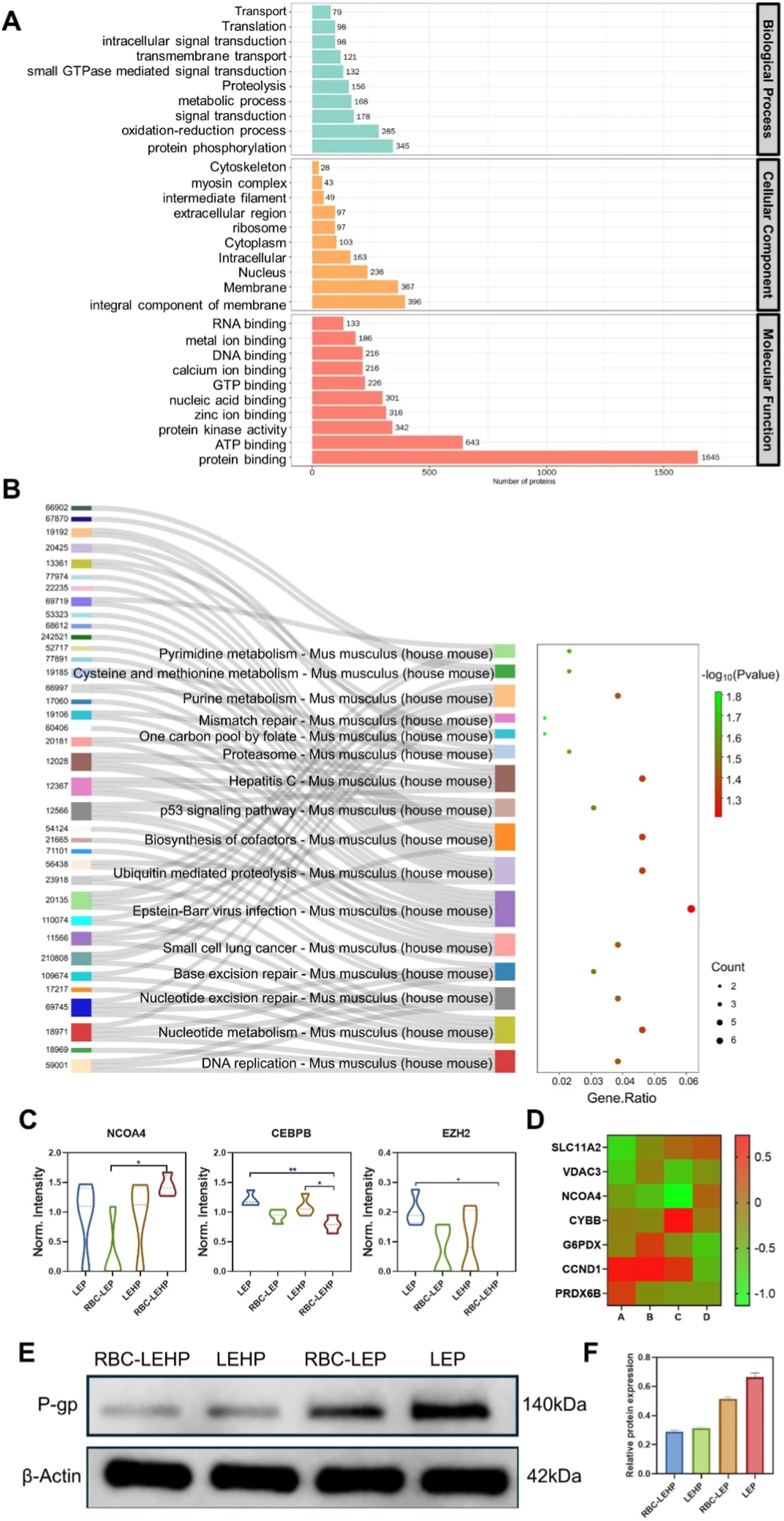
High-throughput proteomic profiling and translational validation of microenvironment reprogramming in pulmonary metastatic tissues. (A) GO functional annotation diagram and (B) KEGG pathway analysis illustratug differentially expressed proteins between the RBC-LEHP and LEP cohorts (n = 4). (C) Violin plots showing the distribution of representative biomarkers and; (D) hierarchical clustering heatmap of differentially expressed proteins associated with hypoxia and ferroptosis across all experimental arms. (E) Representative Western blot bands and(F) corresponding quantification of P-gp expression within lung tissues (n = 3). Data are normalized to *β*-actin loading controls.

At the mechanistic level, the proteomic signatures verified that the RBC-LEHP cascade drives an un-revertable commimtment to ferroptosis (Fig. 9C and D). The selective autophagy receptor NCOA4 was significantly upregulated, confirming the activation of *NCOA4*-mediated ferritinophagy to liberate intracellular releasing free Fe^2+^, which mirrors the labile iron pools and ROS burst observed *in vitro* (Fig. 4). Concurrently, the central antioxidant guardian GPX4 was significantly downregulated, disabling the tumor's lipid peroxide clearance machinery. Beyond iron metabolism, the proteomic data uncovered complementary pathways of tumor suppression: the histone methyltransferase EZH2 - a critical epigenetic driver of cancer stemness and chemoresistance – and the pro-survival transcription factor CEBPB were both markedly suppressed, systematically undermining the tumor's transcriptional adaptation and proliferative capacity.

Consistent with our *in vitro* findings (Fig. 3, Fig. 4), the proteomic data demonstrated that RBC-LEHP profoundly alleviates tumor hypoxia, as evidenced by reduced HIF-1α expression. This hypoxic relief led to a marked downregulation of P-gp (encoded by ABCB1), a key efflux pump that mediates multidrug resistance. The decrease in P-gp was validated by Western blot and provides a direct mechanistic explanation for the enhanced chemosensitivity observed in the RBC-LEHP group (Fig. 9E and F). By lowering P-gp levels, RBC-LEHP restores intracellular paclitaxel accumulation, thereby reversing hypoxia-driven chemoresistance.

These high-throughput proteomic data validate that the RBC-LEHP platform operates via a concerted, multi-mechanistic paradigm: **Hb-driven re-oxygenation** downregulates the HIF-1α/P-gp axis to restore chemosensitivity, **coordinated NCOA4/GPX4 modulation** enforces ferroptotic cell death, and **disruption of the EZH2/CEBPB axis** dismantles epigenetic and transcriptional survival programs, providing a robust biomimetic strategy to eradicate refractory TNBC lung metastases.

## 4 Conclusion

In this study, we successfully engineered a biomimetic, erythrocyte-hitchhiking liposomal system (RBC-LEHP) for the coordinated co-delivery of hemoglobin and paclitaxel to treat metastatic triple-negative breast cancer. By integrating erythrocyte-mediated passive pulmonary targeting and pH-sensitive liposomal tumor internalization, the RBC-LEHP achieves exceptional accumulation within lung metastatic lesions while preserving systemic biosafety.

Mechanistically, the RBC-LEHP platform operates via a highly orchestrated, multi-tiered therapeutic cascade: **Hb-mediated re-oxygenation** downregulates HIF-1α and P-gp to reverse chemoresistance, while simultaneous Fe^2+^ release trigger a Fenton-driven ROS burst that executes robust ferroptotic cell death. This dual-engine mechanism works in profound synergy with the paclitaxel chemotherapy to collapse the tumor's antioxidant and transcriptional survival machinery, resulting in comprehensive tumor regression *in vivo*.

By successfully coupling microenvironment modulation with biomimetic delivery dynamics, this work establishes a sophisticated “targeted delivery → hypoxia alleviation → ferroptosis + chemotherapy → tumor suppression” paradigm. This platform moves beyond conventional single-modality interventions to offer a highly scable and effective approach for overcoming physiological delivery barriers and hypoxia-driven resistance in cancer therapy.

## CRediT authorship contribution statement

**Xunyi You:** Writing – original draft, Investigation. **Kehui Zhu:** Methodology. **Wanjing Li:** Resources. **Hong Wang:** Supervision, Resources. **Rui Zhong:** Resources. **Shen Li:** Resources. **Jiaxin Liu:** Supervision, Project administration. **Yee Shan Wong:** Writing – review & editing. **Subbu S. Venkatraman:** Writing – review & editing. **Ye Cao:** Writing – review & editing, Project administration, Funding acquisition.

## Declaration of competing interest

The authors declare the following financial interests/personal relationships which may be considered as potential competing interests: Ye Cao reports financial support was provided by Sichuan Natural Science Foundation. Ye Cao reports financial support was provided by CAMS Innovation Fund for Medical Sciences. Ye Cao reports financial support was provided by Science and Technology Projects of Xizang Autonomous Region, China. Ye Cao reports financial support was provided by Suzhou Municipal Clinical Medical Expert Team Introduction Program 2026. If there are other authors, they declare that they have no known competing financial interests or personal relationships that could have appeared to influence the work reported in this paper.

## Data Availability

Data will be made available on request.
