# Supplementary material for: Oxygen-augmented erythrocyte-liposome hybrids to overcome paclitaxel resistance in triple-negative breast cancer metastasis via enhanced ferroptosis
Source: Mater Today Bio. 2026 Sep 23;41:103705. doi: 10.1016/j.mtbio.2026.103705 (PMC13636447; doi:10.1016/j.mtbio.2026.103705)
Supplement: Supplementary file 1 [file mmc1.docx]

Supporting Materials

**Table S1.** Liposome characterization (n=3)

|  | LEP | LEHP |
| --- | --- | --- |
| Particle Size（nm） | 168.90±3.36 | 189.17±8.22 |
| Polydispersity Index（PDI） | 0.15±0.01 | 0.14±0.02 |
| Zeta potential (mV) | -3.06±1.22 | -4.97±0.65 |
| Concentration（10^14^ particles/mL） | 1.99±0.20 | 2.65±0.07 |
| Encapsulation efficiency of PTX（%） | 83.32±4.43 | 58.27±2.55 |
| Content of Hb（mg/mL） | / | 0.63±0.05 |

**Table S2.** The combination efficiency of liposomes to RBCs with different incubation ratio (n=3)

| Lipo：RBCs | Encapsulation efficiency of PTX（%） | |
| --- | --- | --- |
|  | RBC-LEP | RBC-LEPH |
| 1μM：3×10^8^ | 51.14±0.62 | 44.91±1.40 |
| 2μM：3×10^8^ | 81.69±1.78 | 67.88±1.05 |
| 3μM：3×10^8^ | 82.57±1.22 | 74.02±5.65 |

**Figure S1.** H&E staining of heart, liver, spleen, and kidney sections from each group.

**Figure S2.** Hemox-Analyzer for assessment of the hemoglobin dissociation curve of LEHP. P50 value-the partial pressure of oxygen at which 50% of the hemoglobin is saturated.
